# Development and validation of the ethnic trust scale in China

**DOI:** 10.3389/fpsyg.2024.1394819

**Published:** 2024-10-14

**Authors:** Yaning Li, Yisheng Yang, Junying Liu, Pai Wang, Zheng Mao

**Affiliations:** ^1^Sciences of Education Department, Baotou Teachers’ College, Baotou, China; ^2^School of Psychology, Inner Mongolia Normal University, Hohhot, China

**Keywords:** China, ethnic trust, conceptual structure, scale development, factor analysis

## Abstract

**Background:**

Considerable research has shown that ethnic trust reflects the existence of friendly relations among all ethnic groups and ethnic individuals, and can help in resolving ethnic conflicts and contradictions, promoting exchanges among various ethnic groups, which is highly relevant to social stability.

**Methods:**

This research, including three studies, aimed to explore the conceptual structure of ethnic trust in China, and develop and validate a measurement of the ethnic trust scale. In the first study, we used free association and in-depth interview methodology, applied cluster analysis, multidimensional scaling analysis, and grounded theory to construct the theoretical framework of Chinese people’s ethnic trust concept. In the second study, we constructed an initial inventory based on the concept dimensions of ethnic trust established in the first study. We screened items by item analysis and extracted common factors using exploratory factor analysis (EFA), thus determining a total of 48 items in the two subscales (interpersonal-oriented ethnic trust subscale and the intergroup-oriented subscale), which consisted of two dimensions including particular trust and universal trust. In the third study, we used first-and second-order confirmatory factor analysis (CFA) to test the scale’s construct validity.

**Results:**

The results indicated a good fit between the two-factor model and the data. And the ethnic trust scale showed very good internal consistency (Cronbach alpha >0.89) and test-retest reliability >0.70.

**Discussion:**

Based on our results, have formed a ethnic trust scale by keeping 48 items, which can beused to measure the levels of interpersonal-oriented and group-oriented ethnic trust within the Chinese cultural context.

## Introduction

From a global perspective, the world today is a multicultural world, with approximately more than 2,500 ethnic groups coexisting on our planet. From an internal perspective of each country, the majority of nations are multicultural nations, where over 2,500 ethnic groups are distributed across more than 200 countries and regions. Many of these countries and regions are faced with the challenge and task of handling ethnic relations effectively. Trust helps to alleviate ethnic conflicts (Parent, 2023), promote exchange and integration among ethnic groups (Tang and Wu, 2020). Given the important role of ethnic trust, this theme has received widespread attention in academia.

Throughout history, Chinese culture has revered harmony, which brings auspiciousness, family cohesion, and national prosperity (Shao and Li, 2001; Wang, 2006; Wang, 2023). As the Analects says, “the use of courtesy, harmony is precious” and “Favorable timing or climate is not as decisive as topographic advantages, and the topographic advantages are not as blessed as the concord arising from the union of people.” in the Mencius. The concept of harmony encompasses both the harmony between humans and nature and the harmony in human relationships (Shao and Li, 2001). The universal value of harmony is the deepest value within China’s mainstream cultural system (Wang, 2023). “Harmony” is also a characteristic of socialist ethnic relations enshrined in our country’s constitution, and it is an important symbol of all ethnic groups in China pursuing a better life and the realization of the “Chinese Dream” together. China is a unified and multi-ethnic country, and harmony is a typical characteristic of the relationship among various ethnic groups in China (Zhao and Zhang, 2008). Trust serves as a fundamental social element for fostering harmonious ethnic relations and is crucial for sustaining social stability through the maintenance of generally harmonious ethnic dynamics (Tang, 2006). Given the unique ethnic characteristics and cultural values of the Chinese nation, Chinese ethnic trust should have its own distinct features. However, its conceptual framework still originates from cross-racial trust abroad. From the perspective of trust subjects, it can be divided into interpersonal trust or intergroup trust. On the one hand, ethnic trust is equivalent to interpersonal trust. Researchers regard it as a stable personality trait, which refers to a universal expectation of the reliability of the words, commitments, and statements of different ethnic members towards their conversation partners. It tends to compare the trait differences in benevolent, integrity, and ability among different ethnic groups (Li and Liang, 2020; Rotter, 1967). It also includes positive interactions among individuals of particular ethnic groups, such as telling the truth and providing support and assistance (Ingelaere, 2016; Li and Liang, 2020; Lv and Ma, 2021; Zhu and Zhou, 2014), exhibiting cross-cultural consistency. On the other hand, researchers equate ethnic trust with intergroup trust, referring to the positive expectations people have towards other groups in intergroup interactions, emphasizing the foundational role of intergroup interactions and identity (Zerfu et al., 2009). In fact, this expectation of positive intergroup interactions is closely related to how individuals construct their self-concept through social categorization. An important premise of self-categorization theory is that people base themselves and others on the salient features of certain social classifications to categorize themselves and others into these social classifications (to obtain social identity) (Tajfel, 1978). This process leads to the development of specific attitudes, emotions, and behaviors. That is, when group identity is activated, depersonality occurs, and people perceive themselves as possessing the cognitive and behavioral characteristics of the group they belong to (Yao et al., 2014), being significantly influenced by the values of the group they belong to (Abrams et al., 2000). People define themselves based on their membership in different social groups. After defining themselves and others as members of the same category, people believed that they are very similar to the attributes of that category and less defined by their unique personal characteristics. In fact, this process is the transition from “I” interpersonal relationships to “we” intergroup relationships. The meaning and research significance of “ethnic group” varies domestically and internationally. For western researchers, “ethnicity” refers to race, and their research targets include local and immigrant ethnic communities (Berning and Ziller, 2017), as well as groups with a history of ethnic conflicts (such as the Greek and Turkish communities in Cyprus, or the Albanian and Serbian communities in Kosovo) (Mironova and Whitt, 2018). The purpose of western research is to mitigate conflicts and rebuild trust. On the other hand, “ethnicity” mainly represents the 56 ethnic groups within the framework of the Chinese nation and the Chinese national community. The purpose of research in China is to strengthen trust and forge the foundation of national unity. These differences also determine that the ethnic trust in China possesses unique characteristics in various ethnic relations, such as the sense of closeness and security through phrases like “the pomegranate blossoms, each seed united” (Cheng, 2020; Li and Wu, 2014), cultural interaction, and intermarriage between ethnic groups (Zhu and Zhou, 2014). From the above results, it can be seen that previous domestic researchers and western researchers are quite similar, often equating ethnic trust with a single dimension of interpersonal or intergroup trust, and rarely considering the unique characteristics of the Chinese ethnicity.

In recent years, Tang and Wu (2020) have integrated two perspectives, viewing ethnic trust as a combination of interpersonal and intergroup trust, expanding the singular conceptual framework of ethnic trust (either interpersonal or intergroup trust) to a dual-dimensional conceptual framework. However, this conceptual framework is not perfect. For instance, while the dimension of interpersonal trust takes into account the interpersonal relationships among acquaintances of various ethnic groups, it lacks an in-depth analysis of the universal trust among members of different ethnicities. Additionally, the dimension of intergroup trust considers the trust among different ethnic groups influenced by policy or legal factors, but it lacks analysis of the particular trust relationships formed by the combination of identity recognition and interactive experiences between different ethnic groups. Therefore, the current conceptual framework of ethnic trust in China is not clear and is urgently in need of improvement.

Compared to the development trajectory of the concept dimension of ethnic trust, the measurement tools are relatively lagging behind. Currently, they only involve tools for measuring interpersonal trust or intergroup trust separately. The interpersonal trust, drawing from the definition by Rotter (1967), often uses questions like “In general, do you think most people/relatives/friends/neighbors in society can be trusted/worthy of trust/good?” for measurement (Li and Ge, 2017). The Interpersonal Trust Scale survey includes interpersonal trust in various situations, involving different social roles (including parents, salespersons, judges, universal public, politicians, and news media). Among them, there are 25 items that cover particular trust factors (trust in peers or other family members) and universal trust factors (trust in unrelated individuals). It uses a Likert five-point self-assessment scale, ranging from 1 to 5 points, with ratings from “strongly agree” to “strongly disagree.” Although this tool has high reliability and validity, it mainly explores the trusting tendencies of trust subjects and neglects the particular content of “ethnicity,” lacking the ethnic characteristics in the Chinese cultural context. The intergroup trust has not yet formed a universally applicable measurement tool. It often adopts modified versions of interpersonal trust measurement tools, such as “The Chinese people are trustworthy/dishonest” statements to measure the level of trust that Americans have in Chinese people (Gries and Crowson, 2010). Additional, combining particular ethnic self-designed tools measure the intergroup trust, such as the trust between Greek Cypriots and Turkish Cypriots through three items (Psaltis, 2011) that “I do not trust Greek Cypriots/Turkish Cypriots because they want to retaliate for what we have done to them in the past,” “When Greek Cypriots/Turkish Cypriots say they love Cyprus, I trust them,” and “When Greek Cypriots/Turkish Cypriots say they want peace, I trust them.” The scale used a Likert 5-point scoring system (1 = strongly disagree, 5 = strongly agree). The content of the scale can reflect the interpersonal trust characteristics of different ethnic groups or the current situation of ethnic trust in the Cyprus region. However, it is difficult to comprehensively explain the concept structure of cross-ethnic trust, and its universality is also limited. Therefore, the intergroup trust measurement tools mentioned above have issues such as a narrow measurement scope (e.g., measuring trust relationships between two nationalities) or simple measurement content (e.g., “Do you think ** people can be trusted?”). Moreover, none of these tools reflect the state of mutual trust between different ethnic groups in China, where “you are in me, and I am in you.” In addition, it is crucial to develop universally applicable to members and groups of all ethnicities in China, while also considering the characteristics of interpersonal and intergroup trust by combining the interpersonal and intergroup characteristics of ethnic trust.

Based on the above thinking, this study intends to explore the dimensions of the public’s view on ethnic trust under the background of Chinese culture through free association and in-depth interviews. It also aims to develop tools and validate their reliability and validity according to psychological measurement standards, with the goal of providing a conceptual analysis framework and effective tools for further exploration of this topic. To achieve the above objectives, we proceed with the following four-steps: (1) identifying the dimensions underlying of ethnic trust in China; (2) generating an initial pool of items and developing an initial scale; (3) exploratory and confirmatory factor analysis to verify the stability of the psychological structure about ethnic trust; (4) reliability and validity testing of the scale.

## Study 1 conceptual structure of ethnic trust

Study 1 used free association and interview parts in succession to identify the dimensions underlying of ethnic trust in China. The free association method is based on the activation-spreading model, which takes into account that concepts in the human mind do not exist in isolation but are interconnected with their “related” concepts, existing in a communal form. Therefore, this study attempts to explore the conceptual connotation of the term “ethnic trust.” By using the free association method to collect words related to the concept of “ethnic trust,” it aims to reveal the intrinsic psychological representation of “ethnic trust” (Liu and Li, 2010). The free association method explores the connotation of the concept of ethnic trust through word categorization, but its results may not be deep enough. Therefore, this study also attempts to use the qualitative research method of semi-structured interviews, which can provide more information value, to further analyze the deep structure of the concept of ethnic trust in the minds of the Chinese public. Combining the two methods to fully and deeply identify the underlying dimensions of the ethnic trust.

### Free association methods

#### Participants

According to the characteristics such as ethnic languages, religious beliefs, and regional distributions, Yang and Wu (2018) divided China’s 56 ethnic groups into five categories, including the Han group, the Manchu-Mongol groups mainly of the Altaic language family, the Tibeto-Burman group of the Sino-Tibetan language family, the Zhuang-Dong or Miao-Yao group of the Southern Sino-Tibetan language family, and the Hui and Uighur groups mainly practicing Islam. This study selected participants from 25 regions, including Inner Mongolia Autonomous Region, Xinjiang Uyghur Autonomous Region, and Guangxi Zhuang Autonomous Region and so on. A total of 729 participants took part in free association of Study 1 and 698 valid questionnaires were received, with an effective rate of 95.75%. This a sample of 698 individuals (female: 331, male: 457; Han ethnic group: 540, Manchu and Mongolian ethnic group: 136, Tibetan ethnic group: 4, Zhuang and Miao-Yao ethnic group: 8, Hui ethnic group: 10; age below 18 years old: 17, aged range18–25: 561, aged range 26–30: 82, aged range 31–40: 23, age above 40 years old: 8; students: 558, teacher: 40, civil servant: 32, laborer: 37), who are used for collecting and categorizing associative words. This study has been approved by the academic ethics committee of a university.

#### Procedure

Six hundred and sixty-seven participants of 698 completed the collecting associative words. In the Free Association procedure, participants engaged in free association around the concept of “ethnic trust” and sequentially wrote down at least five non-repetitive words that best illustrate the concept of “ethnic trust.” In this stage, a total of 3,994 associated words were collected. Another, three participants with ethnic psychology and cultural psychology backgrounds, and skilled in the use of free association techniques adopted a principle of merging and simplifying to combine fully synonymous associations (Meng and Jiang, 2008). Based on this process, 592 meaningfully independent associations related to “ethnic trust” were obtained.

Additionally, 31 participants of 698 independently classified the top 100 words ranked by frequency (cumulative frequency of 3,255, accounting for 81.50% of the total frequency). A systematic clustering and multidimensional scaling analysis were conducted based on the classification results, leading to the formation of an implicit conceptual framework for ethnic trust.

#### Result of free association methods

The results of the system clustering analysis show that the 100 high-frequency words are clustered into four types (see Table 1). The first type includes high-frequency words such as “common progress, common development, shared prosperity, interdependence, share weal or woe, thick and thin together, mutual support, sail on the same tack,” representing the interdependence and solidarity among members of various ethnic groups. The second type includes high-frequency words such as “prosperous, democracy, civilization, harmonious, equality, government by law, legality, openness, unitary,” which these words elaborate on the core values of socialism with Chinese characteristics from the national and social levels, belonging to the domain of group-level values and moral trust. The third type includes high-frequency words such as “friendly, enthusiastic, care, positivist, dedication, struggle, self-improvement, self-confidence, brave, honesty,” representing personal ethical standards. The fourth type includes concepts such as “56 nationalities, regional national autonomy, one China, forging a strong sense of community, pomegranate seeds, family, seek common ground, ethnic intermarriage, all in the family,” which serves as an emotional bond that extends the previously intra-family blood kinship ethics to social, ethnic, and national kinship ethics. The multidimensional scaling analysis further explores their underlying spatial structure. The results indicate that compared to the one-dimensional spatial structure (with stress coefficient Stress = 0.57 > 0.20 and goodness-of-fit coefficient RSQ = 0.21 < 0.80), the fit of the two-dimensional spatial model is better (Stress = 0.10, RSQ = 0.95). Dimension 1 involves intimate emotional interactions among different ethnic groups, such as “share weal or woe, thick and thin together, mutual support, sail on the same tack,” “pomegranate seeds, family, seek common ground, ethnic intermarriage.” It also involves the socialist core values of “individual moral norms” and “social moral norms,” following universal moral judgments, belonging to the ethical trust category. Therefore, the content of Dimension 1 corresponds to the connotation of particular trust and universal trust in social trust. Particular trust refers to trust in acquaintances such as family members, relatives, and neighbors who have blood or geographical relationships; universal trust refers to trust in the general public who have no social relationship. Therefore, this study names Dimension 1 as the familiarity-strangeness relationship closeness dimension, corresponding to special trust and general trust. Likewise, Dimension 2 involves content related to “one China, forging a strong sense of Community of the Chinese nation, family, all in the family, and social moral standards,” respectively reflecting the kinship ethics of the various ethnic groups in China (Liu et al., 2020) and shared values and beliefs (Meng and Wang, 2022), tending to articulate the concept of ethnic trust from a macro intergroup perspective such as the nation and society. On the other hand, ethnic trust includes content such as “common progress, common development, shared prosperity, interdependence” and “personal moral standards,” which belong to the realm of personal moral standards of members of various ethnic groups, representing the inheritance and concrete practice of traditional virtues of the Chinese nation such as “being friends in entering and helping in times of need” and “good virtues do not stand alone, they must have neighbors,” tending to articulate the concept of ethnic trust from an individual perspective. Based on this, this study names Dimension 2 as the interpersonal-intergroup self-categorization dimension.

**Table 1:** The spatial structure of the concept of ethnic trust.

Space area (quadrant)	Dimension naming	Entry classification
Upper left	Interpersonal-particular trust dimension	Common progress, common development, surmoun, co-prosperity, shared prosperity, share weal or woe, thick and thin together, mutual support, Interdependence, sail on the same tack, genuine meeting of minds, love each other devotedly, get on, mutual trust, mutual understanding, mutual appreciation, know each other, mutual learning, coexist, Symbiosis, mutually beneficial, share, Integration, win-win, cooperation, unity, mutual assistance, fraternal love, harmonious
Upper right	Interpersonal-universal trust dimension	Magnanimity, friendly, respect, truthiness, receive, care, communicate, trust, reliant, friendship, freedom, dedication, struggle, elf-improvement, self-confidence, brave, generous, enthusiasm, enjoyment, honesty, cordial, positivity
Lower left	Intergroup-particular trust dimension	Talked different dialects, custom, 56 nationalities, regional national autonomy, national culture, national confidence, The future of the nation, national identity, ethnic relations, national rejuvenation, sibling, verbal communication, cohesion, pomegranate seeds, cultural identity, family, seek common ground, acculturation, ethnic intermarriage, All in the family, ethnic intermingling, community with a shared future, forging a strong sense of community of the Chinese nation, common culture, common language, one china, the Communist Party of China, cultural confidence, sense of belonging
Lower right	Intergroup-universal trust dimension	Government by law, legality, openness, fairness, democracy, prosperous, civilization, just, security, unitary, peace, belief, equality, harmonious, patriotism, gloria

### In-depth interview methods

#### Participants

According to the characteristics of ethnic classification in China (Yang and Wu, 2018), this study selected members of ethnic groups in ethnic gathering areas to ensure the representational of the selected participants as much as possible. For example, when selecting the Altaic language-speaking Manchu and Mongolian ethnic groups, participants were randomly selected from the Mongolian and Manchu ethnic groups relatively concentrated in the Inner Mongolia Autonomous Region of China. Based on the principle of “purposive sampling” (Wu and Huang, 2012), 40 participants were recruited in eight regions from China, including Inner Mongolia Autonomous Region, Guangxi Zhuang Autonomous Region, Ningxia Hui Autonomous Region, and Qinghai Province (Yushu Tibetan Autonomous County) and so on. The number of participants exceeded the minimum sample size requirement of 15 for interview studies (Bertaux, 1981), surpassing the typical sample size of around 30 for most qualitative research studies (Xie and Chen, 2021). In addition to meeting the minimum sample size, saturation is seen as an important criterion in qualitative research to assess whether the sample size is sufficient (Morse, 2015), and is often considered a necessary component of qualitative research methodology. Following the practice of Morse (2015), this study used the method of testing “thematic saturation” to verify the data saturation in qualitative research. The results showed that by the time the 36th interviewee was reached, no new primary codes were added during the coding process, and the state of information saturation was determined by adding 2–3 more individuals. Among the participants, there were Han ethnic group (12 individuals), Mongolian, Manchu ethnic groups (19 individuals), Tibetans ethnic group (2 individuals), Zhuang-Dong and Yao ethnic groups (2 individuals), and Hui ethnic group (5 individuals). The age range was between 18 and 30 years old. The samples’ occupations included college students (30 individuals), civil servants (4 individuals), and employees of other institutions (6 individuals).

#### Procedure

Semi-structured interview qualitative study was used in this survey. The procedure is conducted through one-on-one face-to-face communication, telephone, online audio, and video methods. There is no time limit, and the entire process is recorded. Before the interview, participants carefully read and sign the “Informed Consent Form for Interview.” The interview questions follow the principle of openness, including two parts. The first part is personal information, including understanding the interviewee’s ethnicity and experiences with other ethnic groups. The second part covers issues related to ethnic trust, including 13 questions: (1) How do you understand “ethnic trust”? (2) In what aspects do you think your trust in members of other ethnic groups is manifested? (3) How is ethnic trust formed? What factors influence it? (4) Is your level of trust in other ethnic groups the same as in your own ethnic group? Why? (5) Has your trust in other ethnic groups always remained the same? If there has been a change, please describe the process. (6) In what situations do you lower your trust in other ethnic groups? Why? (7) What do you think are the main reasons that could lead to a lower level of ethnic trust? (8) How can we start from which aspects to improve ethnic trust levels? What particular actions can be taken? (9) Please share with me someone from another ethnic group whom you trust and what qualities make you trust them? (10) How has mutual trust between different ethnic groups changed your life? Can you elaborate? (11) How do you think future ethnic relations will change? Why? (12) What similarities and differences do you see between ethnic trust and other types of trust? (13) What do you think is the relationship between trust among China’s various ethnic groups and trust in other countries? Why? The interview data is transcribed verbatim and organized into 40 interview texts, with a cumulative time of 3,521 min and a total of 759,596 characters.

Based on grounded theory (Flick, 2007), the Nvivo12.0 plus is used to conduct content analysis of the interview texts using open coding, axial coding, and selective coding procedures (Chen, 2000; Wu and Huang, 2012; Zhang and Yang, 2018). First, two members of the ethnic psychology research team participated in the coding process. Corresponding identifiers were set for all interviewees (such as FC01 representing the first female urban individual, and MR10 representing the 10th male rural individual) to avoid confusion in later data analysis stages, laying the foundation for data analysis (Chen, 2000). Coders maintained an open mindset, attempted to suspend personal biases and the consensus of the research community, and carefully considered the content of words, sentences, and paragraphs imported into the data using N12plus software, with “ethnic trust” as the core topic. By searching for repeatedly occurring meaningful units in the interviewee data, meaningful logging codes were extracted for the research problems, and the data were coded. In the open coding process, we used the interviewees’ own expressions as much as possible to allow initial concepts to naturally emerge. For example, “whether in Chinese law or in our common consciousness, thoughts, and ideas, mutual equality has become a common one among us, like a common sense” that can be coded as “mutual equality.” Second, the axial coding process refined, adjusted, and categorized the initial concepts extracted during the open coding process, merged parts with similar or related meanings, and clarified, organized the inherent connections between the codes (Flick, 2007), thus forming main categories, linking main categories and subcategories for relevancy, comparing different categories to create an organically linked whole of the data (Wu and Huang, 2012; Zhang and Yang, 2018). Third, selective coding involved handling the data from spindle coding at a more abstract level, aiming to consolidate developed categories and explaining the major category by connecting these subcategories, shaping the stories of each case (Flick, 2007), and simultaneously measuring and determining multiple important phenomena, eventually generating several main categories and their associated subcategories.

#### Result of in-depth interview methods

##### The three-level coding results of the grounded theory

Based on the grounded theory (Flick, 2007), this research extracted a total of 737 reference points, 12 initial concepts, 4 main axis nodes, and 1 core code (see Table 2) particularally: During the process of comparative analysis, the researcher found that the codes “not being excluded,” “not being particular,” and “no need to worry about ethnic labels” in open coding had consistency in the data, so these 3 codes were classified into the category of “treating all attitudes equally.” On the other hand, the data related to codes such as “peaceful coexistence,” “non-interference,” “non-offense,” and “let it be” were different from those in the category of “treating all attitudes equally,” thus these 4 codes were classified into the category of “principles of peaceful coexistence.” In total, there were 737 reference points involved. Next, focusing on “ethnic trust,” by comparing the intrinsic relationships of 12 initial concepts, they were summarized into 4 more refined main axis nodes. Finally, by further summarizing the inherent relationships between the main axis nodes, the core category is formed, which is the concept structure of ethnic trust.

**Table 2:** Continuous comparison: a comparison of the conceptual structures of ethnic trust obtained by in-depth interview method and free association method.

Main axis nodes (reference point)	Initial concepts (reference point)	A comparison result of the in-depth interview method and free association method
Intergroup universal trust (197)	Perception of fairness and justice (117)	Legality, just, equality, unitary, security
	Common societal values (24)	government by law, patriotism, prosperous, civilization, democracy, harmonious, gloria, belief
	Peaceful association (56)	Peace
Intergroup particular trust (341)	Ethnic positive emotions (19)	All in the family, community with a shared future, one china, the Communist Party of China, sense of belonging, ethnic relations, sibling, cohesion, pomegranate seeds, family
	Culture exchange and symbiosis (300)	Fifty-six nationalities, regional national autonomy, national culture, national confidence, The future of the nation, national identity, custom, cultural identity, national rejuvenation, talked different dialects, verbal communication, acculturation, seek common ground, cultural confidence, common language, common culture, forging a strong sense of community of the Chinese nation, ethnic intermingling, ethnic intermarriage
	Economic interconnection and mutual assistance (22)	(Unique results of qualitative research)
Interpersonal universal trust (57)	Benevolent-warmth perception (22)	Friendly, cordial, enthusiasm, magnanimity, care, respect, truthiness, receive, communicate, trust, friendship, freedom
	Integrity-morality perception (13)	Honesty, reliant, adhere faithfully
	Ability-competence perception (22)	Self-improvement, confidence, dedication, struggle, self-brave, positivity, generous, enjoyment
Interpersonal particular trust (142)	Interpersonal emotional reciprocity (22)	Genuine meeting of minds, love each other devotedly, mutual trust, mutual understanding, mutual appreciation, know each other, mutual learning
	Close relationships and mutual assistance (64)	Fraternal love, get on, harmonious, mutual assistance, mutually beneficial, symbiosis, coexist, share, integration, win-win, co-prosperity, shared prosperity, share weal or woe, common progress, common development, surmoun, cooperation, unity, hick and thin together, mutual support, sail on the same tack
	Tendency to express emotions (56)	(Unique results of qualitative research)

Among them, the 12 initial concepts specifically include the perception of fairness and justice, indicating that individuals of all nationalities in China will not worry about being treated differently due to differences in language, lifestyle, and other ethnic group characteristics, and have the perception of equality in terms of legal status, rights, obligations. The common societal values refer to the common value orientation formed in the ethical practices of interactions among various ethnic groups in China. The peaceful association refers to the relationship among various ethnic groups in China that follow the principle of peaceful coexistence, do not discriminate, exclude, isolate, or offend each other. The ethnic positive emotions refer to the positive feelings, sense of belonging, and dependence that people have towards other ethnic groups with whom they have long-term interactions. The culture exchange and symbiosis refer to the behavior of people willing to engage in cultural contact, collision, mutual learning, and mutual absorption with cross-ethnic groups. The economic interconnection and mutual assistance refer to the mutual dependence, mutual assistance, and common development of national friendly relations formed in the economy with other ethnic groups. The interpersonal emotional reciprocity refers to the emotional connection between members of different ethnic groups and other ethnic groups with blood or geographical connections, such as treating each other sincerely among family members, relatives, neighbors, and acquaintances, providing a sense of security and support. The close relationship and mutual assistance refers to the behavior of people getting close to and helping each other with familiar members of their own ethnic group. The tendency to express emotions refers to people sharing their secrets, life events, emotional experiences, and other information with familiar members of their own ethnic group. The benevolent-warmth perception refers to the perception results of people’s ability to exclude self-interest motives and voluntarily provide help to communication partners. The integrity-morality perception refers to the perception results of people’s reliability and credibility towards communication partners. The ability-competence perception refers to the judgment results of people’s assessment of the skills and abilities held by communication partners.

According to the above concepts, the subjects of the perception of fairness and justice, the common societal values, and the peaceful association are all various ethnic groups, representing a general trust view among ethnic groups that do not have specific social relationships. Similarly, the subjects of the ethnic positive emotions, the culture exchange and symbiosis, and economic interconnection and mutual assistance are also various ethnic groups, but these three initial concepts more involve the trust in familiar ethnic groups. For example, the interviewer mentioned that “ethnic trust is a sense of dependence that one’s own ethnic group and other ethnic groups develop in learning and life.” The subject of the interpersonal emotional reciprocity, the close relationship and mutual assistance, and the tendency to express emotions are the members of various ethnic groups. It is the trust relationship established by people at the interpersonal level and with specific members of other ethnic groups, as mentioned in the interviewers “Anyway, when I feel sad, I would express it once I enter the dormitory (which is a multi-ethnic mixed dormitory).” Similarly, the benevolent-warmth perception, the integrity-morality perception, and the ability-competence perception also belong to the interpersonal level and are a common trust in the public. Based on the above analysis, the 12 initial concepts are classified into 4 main code axes, namely the intergroup-universal trust (3 initial concepts, 197 reference points) and the intergroup-particular trust (3 initial concepts, 341 reference points), the interpersonal-particular trust (3 initial concepts, 142 reference points) and the interpersonal-universal trust (including 3 initial concepts, 57 reference points).

### Result of Study 1

**Figure 1:**
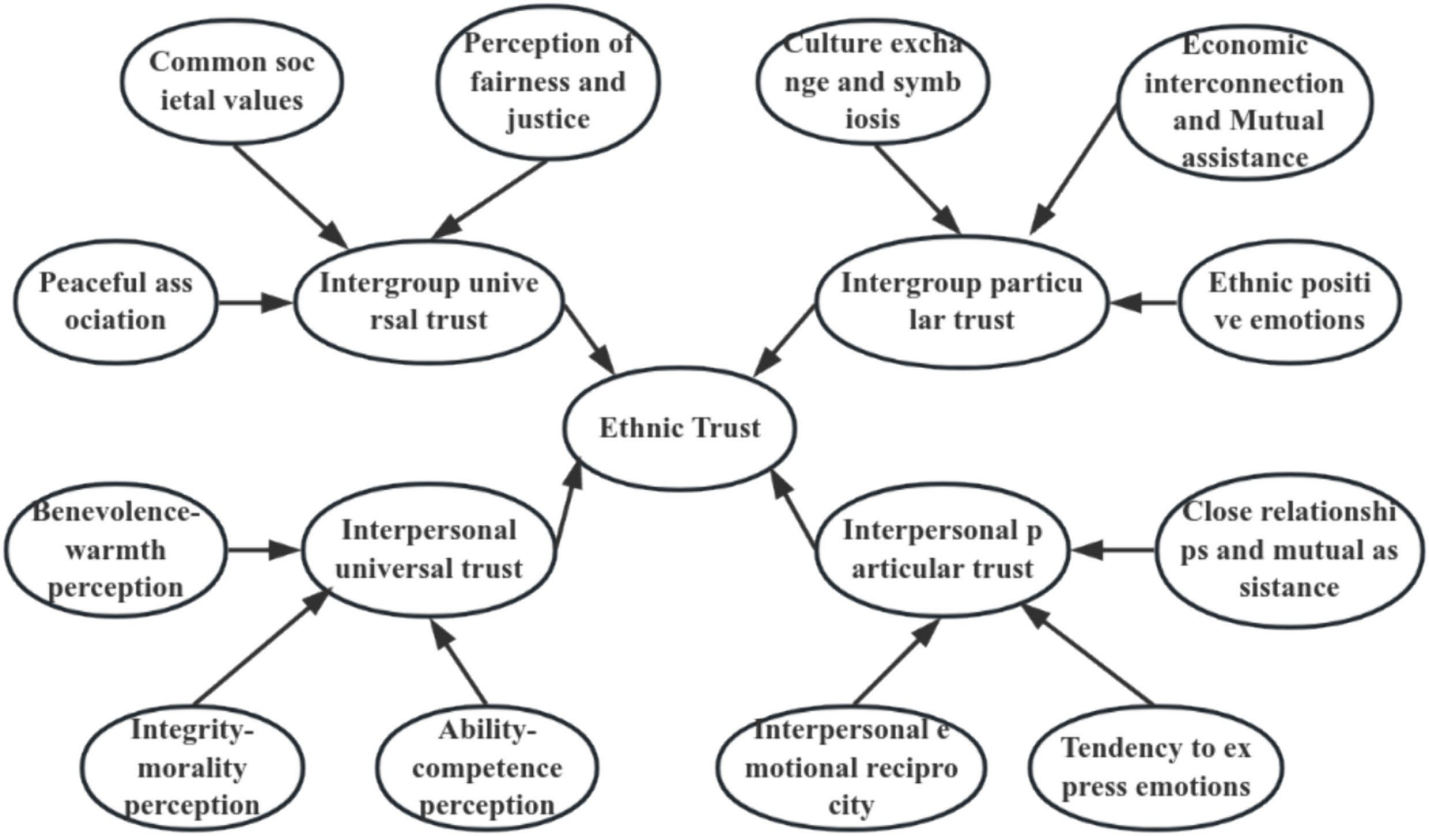
Dimensional construction diagram of the concept of ethnic trust.

Based on the results of free association and in-depth interviews, the conceptual structure of ethnic trust can be preliminarily constructed into four dimensions: interpersonal universal trust, interpersonal particular trust, intergroup universal trust, and intergroup particular trust (see Table 2 and Figure 1).

The conceptual structure of ethnic trust in china is not only in line with the dimensions of interpersonal and intergroup trust mentioned by (Tang and Wu, 2020), but also innovative, where both interpersonal and intergroup dimensions consist of factors of universal trust and particular trust.

## Study 2 development of the ethnic trust scale

Building on the foundation of exploring the concept structure of ethnic trust through free association and in-depth interview methods in Study 1, Study 2 explores and verifies the concept structure of ethnic trust by compiling corresponding questions and developing a Chinese localized scale of ethnic trust.

### Methods

#### Participants

Study 2 used stratified sampling as the primary method and snowball sampling as a supplementary method to obtain sample information. The stratified sampling involved the regions of Inner Mongolia, Northeast China, North China, East China, etc., involving 28 provinces and regions including Inner Mongolia Autonomous Region, Guangxi Zhuang Autonomous Region, Xinjiang Uygur Autonomous Region, Tibet Autonomous Region, etc. A total of 1,022 participants took part in this study and 899 valid questionnaires were received, with an effective rate of 87.96%. Hundred and twenty-three participants (12.04%) were excluded from the analysis because they did not pass the quality control questions, impression management questions (both questions were selected as “strongly agree”), and their questionnaires with most or all answers being the same. This resulted in 899 participants (female: 470, male: 429; Han ethnic group: 693, Manchu and Mongolian ethnic group: 104, Tibetan ethnic group: 7, Zhuang and Miao-Yao ethnic group: 37, Hui ethnic group: 58; age below 18 years old: 6, aged range 18–25: 669, aged range 26–30: 167, aged range 31–40: 38, age above 40 years old: 19; high school education or below: 47, associate degree/vocational college: 122, undergraduate: 681, master’s degree or above: 42; civil servant: 23, public institution staff: 77, company staff: 96, self-employed individual/entrepreneur: 104, laborer: 88, students: 513), who are used for exploratory factor analysis. All participants claimed to be free of current and previous neurological and psychiatric disorders and were not currently using psychotropic medication.

#### Procedure

A three-step approach was used to develop the Ethnic Trust Scale: 1. Item generation; 2. Item reduction; 3. Exploration of scale structure.

#### Item generation

The basic framework for the design of the questionnaire in this study is: Ethnic Trust → Main Dimensions → Sub Dimensions → Measurement Indicators → Specific Questions/items (see Table 3). Specifically, the main dimensions to be included in this measurement questionnaire are first determined. Due to Leary and Tangney (2003) believe that personal identity and social identity are mutually exclusive and cannot be highlighted at the same time, the ethnic trust has two main dimensions relatively independently, which are named the interpersonal-oriented ethnic trust and the intergroup-oriented ethnic trust. Therefore, we attempted to develop two sub-scales named the interpersonal-oriented ethnic trust scale and the intergroup-oriented ethnic trust scale. In each main dimension, it is further operationalized into “universal trust” and “particular trust” as two sub-dimensions. Then, each sub-dimension is further operationalized into several specific indicators/items. Items for the interpersonal ethnic trust and intergroup ethnic trust were not only selected by use the wording from interviews or free association test, but also referring to the Interpersonal Trust Scale (Rotter, 1967), the National Unity Consciousness Scale (Chen and Fan, 2021), the Ethnic Fusion Attitude Scale (Chen and Xue, 2022), and the Chinese National Community Awareness Scale (Chen and Xue, 2021). As a result, a scale with 266 items was developed. Each item is scored using a 7-point scale (1 = “strongly disagree,” 4 = “uncertain,” 7 = “strongly agree”).

**Table 3:** The process of operationalizing the concept of ethnic trust and its specific indicators/items.

Concept	Main dimensions	Sub-dimensions	Measurement indicators/items
Ethnic trust	Intergroup trust	Universal trust	Perception of fairness and justice, common societal values, ethnic positive emotions
		Particular trust	Ethnic positive emotions, culture exchange and symbiosis, economic interconnection and mutual assistance
	Interpersonal trust	Universal trust	Benevolent perception, integrity perception, ability perception
		Particular trust	Interpersonal emotional reciprocity, close relationships and mutual assistance, tendency to express emotions

### Item reduction

Twelve psychology graduate students reviewed and evaluated the relevance and clarity of expression of the items (formula: average rank/total number of ranks), as well as the occurrence of item redundancy (Colquitt et al., 2019). Items with relevance and clarity values below 0.6 and redundant semantically repeated items were deleted, leaving 130 items. Finally, two impression management questions and two lie detection questions were included (Zhong et al., 2021), totaling 134 questions. Due to the mutual exclusivity of personal and social identities, the two identities cannot be highlighted at the same time (Leary and Tangney, 2003). These 134 questions are divided into two sub-scales: 65 items are about interpersonal trust orientation of ethnic trust, and 69 items are about intergroup trust orientation of ethnic trust. All items are scored using a 7-point scale (1 = “strongly disagree,” 4 = “uncertain,” 7 = “strongly agree”), with all items scored in a positive direction.

### Exploration of scale structure

Eight hundred and ninety-nine participants completed the initially developed interpersonal trust orientation questionnaire and intergroup trust orientation questionnaire of the ethnic trust, totaling 134 items. SPSS 25.0 software was used to project analysis and exploratory factor analysis. Construct validity was assessed using exploratory factor analysis to determine the key components of the 65-item questionnaire and the 69-item questionnaire.

### Result

#### Item analysis

Before conducting the Exploratory Factor Analysis (EFA), this study utilized item analysis (Wu, 2010) to preliminarily select items and checked the data file to ensure it met the necessary conditions for analysis, checking for errors or missing values.

After excluding the polygraph item, 130 items were screened by item analysis. Firstly, the data of interpersonal orientation and intergroup orientation of ethnic trust were separately divided into high and low groups (27%) for independent-samples *t*-test by using the critical ratio method. The criteria for deletion were as follows: (1) The critical value was not significant (*p* > 0.05); (2) The *t* statistic of the difference between high and low item groups was lower than 3 (*t* < 3). The results showed that all items were up to standard, so they were retained without deleted (Wu, 2010).

Furthermore, correlations between item scores and total scores of interpersonal orientation and intergroup orientation of ethnic trust were examined. Based on Pearson correlation coefficients, criteria for deletion were: (1) Non-significant correlations with the total scale (*p* > 0.05); (2) correlation coefficients (*r*) between item scores and total scores below 0.4 or above 0.8. The results indicated that item1 did not meet the criteria and was therefore removed (see Table 1A and Table 2B are in the item analysis of Supplementary material).

Combined with the above item analysis methods, 1 item were deleted and 129 items were retained.

#### Exploratory factor analysis

To analysis an *a priori* subdivision of the items’ pool to adequately fit the structure of the four factors of ethnic trust, we first conducted an exploratory factor analysis (EFA) on the overall pool of items, and then conducted exploratory factor analysis separately for interpersonal-oriented ethnic trust and intergroup-oriented ethnic trust.

The Kaiser–Meyer–Olkin (KMO) value (KMO = 0.98) and Bartlett’s Test of Sphericity (χ^2^_ethnic trust_ = 35095.68, *df* = 1,128, *p* < 0.001; χ^2^_interpersonal trust_ = 52553.05, *df* = 251, *p* < 0.001; χ^2^_intergroup trust_ = 66503.04, *df* = 251, *p* < 0.001) showed that the items of this scale were appropriate for factor analysis. In our study, 899 valid samples were retained, which met the minimum number of sample observations for each variable (Stevens, 2002). Moreover, PCA and Varimax were used to analyze 129 items. The items that did not meet the standard and theoretical expectation were deleted. The criteria for deletion were as follows: (1) The factor loading was lower than 0.45; (4) The item appeared in two or more factors at the same time; (5) There were only 1–2 items in the factors. As a result, 81 items were deleted.

Then, the remaining 48 items were used for EFA. Four factors of ethnic trust and two factors of interpersonal orientation and intergroup orientation of ethnic trust were separately emerged with eigenvalues larger than 1, with a cumulative variance interpretation rate of 63.91 and 64.34% (see Tables 4–6). All items’ communalities ranged from 0.53 to 0.73, and factor loadings ranged from 0.50 to 0.80.

**Table 4:** Exploratory factor analysis results of the EthnicTrust Scale (*n* = 899).

Items	Factor loading				Communalities
	Interpersonal universal trust	Interpersonal particular trust	Intergroup universal trust	Intergroup particular trust	
No matter which ethnicity, the vast majority of people are friendly.	0.59				0.62
I can usually feel the benevolent naturally expressed by people of all ethnic groups.	0.64				0.69
I think that the majority of people from different ethnic groups are quite diligent in their work.	0.64				0.68
I think that honesty is the best quality for people of any ethnicity.	0.61				0.61
Most people of all ethnic groups are trustworthy in keeping their promises.	0.67				0.71
I believe every ethnic group has excellent individuals in a certain professional field.	0.66				0.66
I believe that the majority of people in all ethnic groups are capable of doing their own work well.	0.69				0.70
I am willing to cooperate with capable people regardless of their ethnicity.	0.65				0.71
When others have strong professional skills, I do not mind their ethnic identity information.	0.56				0.59
If I entrust important matters to cross-ethnic relatives, friends, neighbors, or other acquaintances, I do not need to explain and closely supervise repeatedly.		0.69			0.61
I will be very relieved to hand things over to a cross-ethnic acquaintance for handling.		0.71			0.66
When facing difficulties and setbacks, I can receive care, understanding, or encouragement from friends of different ethnic groups.		0.70			0.68
I often confide my troubles with my close friends from different ethnic groups.		0.69			0.64
I can confide secrets to relatives, friends, neighbors, or colleagues of different ethnicities.		0.75			0.63
Besides friends from my own ethnic group, I also want to share happy with friends of different ethnicities as soon as possible (such as good news in life).		0.70			0.66
In front of trusted cross-ethnic members, I will not hide my true thoughts.		0.71			0.67
I believe that acquaintances such as cross-ethnic relatives, friends, neighbors, or colleagues will not joke with me maliciously.		0.74			0.64
I and cross-ethnic relatives, friends, neighbors, or colleagues and other acquaintances, will not misinterpret each other’s intentions when joking.		0.72			0.65
Sometimes when I communicate with acquaintances (such as relatives, friends, neighbors or colleagues) from different ethnic backgrounds, I naturally use a joking tone to express my thoughts and feelings.		0.73			0.62
Cross-ethnic relatives, friends, neighbors, colleagues, and other acquaintances often invite me to share food.		0.72			0.63
In daily life, I am willing to accompany acquaintances from different ethnic groups.		0.66			0.66
When I need help, I believe that relatives, friends, neighbors, or colleagues from different ethnic groups will do their best to help me.		0.68			0.52
When I ask for help from friends of different ethnic backgrounds, I am not worried about causing trouble for them.		0.71			0.66
When help is needed, I will actively seek help from other trusted members of different ethnic backgrounds.		0.71			0.57
My attitude towards my own ethnic and other ethnic is the same.			0.57		0.57
All ethnic group have the right to equal participation in the political life of our country.			0.63		0.60
Every ethnic group will consciously safeguard the unity of the motherland and oppose secession.			0.75		0.67
I am not afraid to reveal or show my ethnicity in public settings.			0.67		0.60
I believe that the 56 ethnic groups in China can live together harmoniously.			0.76		0.71
The core socialist values are an important bond for maintaining trust between the 56 ethnic groups in the country.			0.75		0.69
The core socialist values are common spiritual norms shared by all ethnic groups.			0.77		0.71
The Chinese cultural concept of “Harmony under Heaven” binds together various ethnic groups as one.			0.74		0.70
Integrity is moral standards that all ethnic groups need to abide by.			0.76		0.72
The great rejuvenation of the Chinese nation is a common value guidance for all ethnic groups.			0.78		0.73
When facing external threats, the 56 ethnic groups in China can unite.			0.73		0.69
When interacting with familiar ethnic groups, I cannot help but see us as one entity.				0.51	0.60
All ethnic groups work together and support each other in economic development.				0.52	0.61
I believe that familiar cross-ethnic groups will not intentionally violate my ethnic taboos.				0.50	0.56
Compared to unfamiliar groups, I can better understand the unique cultures of other familiar ethnicities.				0.70	0.61
When other ethnic groups use their unique languages, I believe it is simply because they are more proficient in their own languages.				0.69	0.61
I like to share books, videos, and other materials related to my own ethnic culture with other ethnic groups that I am familiar with.				0.71	0.69
I am very willing to listen to people from other ethnic groups share the unique aspects of their ethnicities (such as cultural customs, etc.).				0.59	0.67
I am willing to deeply communicate with other ethnic groups about the cultures customs of our respective ethnicities.				0.62	0.60
I will consider the different customs of my own ethnic group and other ethnic groups as distinctive cultural features of each other.				0.60	0.66
I am willing to actively learn about the unique cultures of other ethnic groups.				0.65	0.61
Compared to unfamiliar groups, I prefer to embrace familiar cross-cultural customs.				0.70	0.64
I am willing to trust the familiar ethnic group because our cultures blend more.				0.68	0.63
Universally, people will not reject interethnic marriage if they have a high level of trust in other ethnic groups.				0.60	0.57
Eigenvalues	1.319	22.771	4.689	2.150	
Contribution rate (%)	2.748	47.439	9.769	4.479	
Cumulative contribution rate (%)	2.748	50.187	59.956	64.435	

**Table 5:** Exploratory factor analysis results of the Interpersonal Trust Scale in Different Ethnic Groups (*n* = 899).

Items	Factor loading		Communalities
	Interpersonal universal trust	Interpersonal particular trust	
No matter which ethnicity, the vast majority of people are friendly.	0.69		0.62
I can usually feel the benevolent naturally expressed by people of all ethnic groups.	0.71		0.67
I think that the majority of people from different ethnic groups are quite diligent in their work.	0.67		0.64
I think that honesty is the best quality for people of any ethnicity.	0.76		0.63
Most people of all ethnic groups are trustworthy in keeping their promises.	0.73		0.69
I believe every ethnic group has excellent individuals in a certain professional field.	0.78		0.66
I believe that the majority of people in all ethnic groups are capable of doing their own work well.	0.75		0.68
I am willing to cooperate with capable people regardless of their ethnicity.	0.80		0.72
When others have strong professional skills, I do not mind their ethnic identity information.	0.71		0.60
If I entrust important matters to cross-ethnic relatives, friends, neighbors, or other acquaintances, I do not need to explain and closely supervise repeatedly.		0.68	0.60
I will be very relieved to hand things over to a cross-ethnic acquaintance for handling.		0.71	0.63
When facing difficulties and setbacks, I can receive care, understanding, or encouragement from friends of different ethnic groups.		0.70	0.68
I often confide my troubles with my close friends from different ethnic groups.		0.70	0.63
I can confide secrets to relatives, friends, neighbors, or colleagues of different ethnicities.		0.75	0.63
Besides friends from my own ethnic group, I also want to share happy with friends of different ethnicities as soon as possible (such as good news in life).		0.70	0.65
In front of trusted cross-ethnic members, I will not hide my true thoughts.		0.69	0.65
I believe that acquaintances such as cross-ethnic relatives, friends, neighbors, or colleagues will not joke with me maliciously.		0.74	0.67
I and cross-ethnic relatives, friends, neighbors, or colleagues and other acquaintances, will not misinterpret each other’s intentions when joking.		0.74	0.63
Sometimes when I communicate with acquaintances (such as relatives, friends, neighbors or colleagues) from different ethnic backgrounds, I naturally use a joking tone to express my thoughts and feelings.		0.75	0.65
Cross-ethnic relatives, friends, neighbors, colleagues, and other acquaintances often invite me to share food.		0.72	0.61
In daily life, I am willing to accompany acquaintances from different ethnic groups.		0.66	0.63
When I need help, I believe that relatives, friends, neighbors, or colleagues from different ethnic groups will do their best to help me.		0.68	0.65
When I ask for help from friends of different ethnic backgrounds, I am not worried about causing trouble for them.		0.72	0.53
When help is needed, I will actively seek help from other trusted members of different ethnic backgrounds.		0.71	0.64
Eigenvalues	1.785	11.96	
Contribution rate (%)	7.439	49.832	
Cumulative contribution rate (%)	7.439	57.271	

**Table 6:** Exploratory factor analysis results of the Intergroup Trust Scale in Different Ethnic Groups (*n* = 899).

Items	Factor loading		Communalities
	Intergroup universal trust	Intergroupparticular trust	
My attitude towards my own ethnic and other ethnic is the same.	0.62		0.56
All ethnic group have the right to equal participation in the political life of our country.	0.68		0.60
Every ethnic group will consciously safeguard the unity of the motherland and oppose secession.	0.76		0.67
I am not afraid to reveal or show my ethnicity in public settings.	0.71		0.59
I believe that the 56 ethnic groups in China can live together harmoniously.	0.79		0.71
The core socialist values are an important bond for maintaining trust between the 56 ethnic groups in the country.	0.78		0.69
The core socialist values are common spiritual norms shared by all ethnic groups.	0.79		0.72
The Chinese cultural concept of “Harmony under Heaven” binds together various ethnic groups as one.	0.75		0.70
Integrity is moral standards that all ethnic groups need to abide by.	0.78		0.72
The great rejuvenation of the Chinese nation is a common value guidance for all ethnic groups.	0.78		0.71
When facing external threats, the 56 ethnic groups in China can unite.	0.74		0.69
When interacting with familiar ethnic groups, I cannot help but see us as one entity.		0.54	0.57
All ethnic groups work together and support each other in economic development.		0.55	0.59
I believe that familiar cross-ethnic groups will not intentionally violate my ethnic taboos.		0.75	0.55
Compared to unfamiliar groups, I can better understand the unique cultures of other familiar ethnicities.		0.75	0.60
When other ethnic groups use their unique languages, I believe it is simply because they are more proficient in their own languages.		0.69	0.61
I like to share books, videos, and other materials related to my own ethnic culture with other ethnic groups that I am familiar with.		0.77	0.68
I am very willing to listen to people from other ethnic groups share the unique aspects of their ethnicities (such as cultural customs, etc.).		0.66	0.67
I am willing to deeply communicate with other ethnic groups about the cultures customs of our respective ethnicities.		0.67	0.61
I will consider the different customs of my own ethnic group and other ethnic groups as distinctive cultural features of each other.		0.63	0.64
I am willing to actively learn about the unique cultures of other ethnic groups.		0.70	0.60
Compared to unfamiliar groups, I prefer to embrace familiar cross-cultural customs.		0.76	0.64
I am willing to trust the familiar ethnic group because our cultures blend more.		0.73	0.63
universally, people will not reject interethnic marriage if they have a high level of trust in other ethnic groups.		0.67	0.56
Eigenvalues	11.806	2.184	
Contribution rate (%)	49.193	9.10	
Cumulative contribution rate (%)	49.193	58.292	

The overall item pool can be divided into four factors (see Table 4), which lays the foundation for the factor subdividing *a priori* between interpersonal and intergroup trust.

In interpersonal orientation of ethnic trust (see Table 5), factor 1 was named “interpersonal universal trust,” which referred to trust that the trust in strangers from other ethnic groups with whom there is no existing social relationship. This factor is composed of 9 items, including the benevolent perception, integrity perception, and ability perception. The benevolent perception that referred to the recognition of positive traits in others, such as the ability to set aside self-interest and offer help voluntarily, akin to the perception of benevolence and compassion. The integrity perception referred to that the recognition of the reliability and consistency in the words and actions of the people one interacts with. The ability perception referred to that the judgment of the skills and abilities that people attribute to the individuals they interact with. Factor 2 was named “interpersonal particular trust,” which referred to trust that the trust people have in acquaintances from other ethnic groups who have blood or geographical ties, such as family members, relatives, neighbors, and classmates. This factor is composed of 15 items, including interpersonal emotional reciprocity, close relationships and mutual assistance, and tendency to express emotions. The interpersonal emotional reciprocity referred to that the intimate relationships where people, with sincerity, provide a sense of security and emotional support to acquaintances from other ethnic groups who share blood or geographical ties, such as family, relatives, neighbors, and classmates. The close relationships and mutual assistance referred to that the actions of people being close to and helping familiar members of other ethnic groups. The tendency to express emotions referred to that the motivation of individuals to share their secrets, life events, and emotional experiences with familiar members of other ethnic groups.

In intergroup orientation of ethnic trust (see Table 6), factor 1 was named “intergroup universal trust,” which referred to trust in cross-ethnic groups that do not have any social relationships (other ethnic groups within the country besides one’s own). This factor is composed of 11 items, including the specific content of peaceful association, common societal values, and perception of fairness and justice. The peaceful association referred to that the relationships among all ethnic groups in China that adhere to the principles of peaceful coexistence, where there is no discrimination, exclusion, isolation, or offense against any ethnic group. Common societal values referred to that the common value orientations formed by all ethnic groups in China through their ethical practices in mutual interactions. The perception of fairness and justice refers to that the belief among all ethnic groups in China that they will not be treated differently due to differences in language, living habits, and other ethnic group characteristics, and that they enjoy equal perception in terms of legal status, rights, and obligations. Factor 2 was named “intergroup particular trust,” which referred to trust that individuals cultivate with other ethnic groups with whom they share close relationships, often through familial bonds, geographical connections, and similar ties. This factor is composed of 13 items, including the culture exchange and symbiosis, economic interconnection and mutual assistance, and ethnic positive emotions. The culture exchange and symbiosis referred to that the readiness of individuals to engage in cultural contact, interaction, mutual learning, and reciprocal absorption with cross-ethnic groups. The economic interconnection referred to that the friendly ethnic relations formed between people and other ethnic groups, characterized by mutual economic reliance, assistance, and collective development. The ethnic positive emotions referred to that the positive feelings such as affinity, reliance, and closeness that individuals develop through long-term interactions with cross-ethnic groups.

## Study 3 validation of the ethnic trust scale

In Study 3, the modified ethnic trust scale was used to survey another sample of 671 multiethnic individuals. Confirmatory factor analyses were used to identify the possible dimensions of ethnic trust scale and then to calculate the construct validity, criterion-related validity, internal consistency reliability and test–retest reliability (Lu et al., 2009), and fit indices. The results of this analysis were used to further explore and construct the latent factor structure of the ethnic trust and the subset of items with the cleanest factor loadings.

### Methods

#### Participants

Study 3 used stratified sampling as the primary method and snowball sampling as a supplementary method to obtain sample information. The stratified sampling involved the regions of Inner Mongolia, Northeast China, North China, East China, etc., involving 31 provinces and regions including Inner Mongolia Autonomous Region, Guangxi Zhuang Autonomous Region, Xinjiang Uygur Autonomous Region, Tibet Autonomous Region, etc. A total of 900 participants took part in this study and 671 valid questionnaires were received, with an effective rate of 74.6%. Two hundred and twenty-nine participants (25.4%) were excluded from the analysis because they did not pass the quality control questions, impression management questions (both questions were selected as “strongly agree”), and their questionnaires with most or all answers being the same. This resulted in 671 participants (female: 339, male: 342, Han ethnic group: 557, Manchu and Mongolian ethnic group: 53, Tibetan ethnic group: 10, Zhuang and Miao-Yao ethnic group: 28, Hui ethnic group: 23; age below 18 years old: 7, aged range 18–25: 456, aged range 26–30: 143, aged range 31–40: 53, age above 40 years old: 12; high school education or below: 33, associate degree/vocational college: 70, undergraduate: 480, master’s degree or above: 88; civil servant: 14, public institution staff: 45, company staff: 111, self-employed individual/entrepreneur: 33, laborer: 16, students: 396), who are used for exploratory factor analysis. All participants claimed to be free of current and previous neurological and psychiatric disorders and were not currently using psychotropic medication.

Due to the goal sample size of effect size association validity is determined by the calculation result of G*Power (Faul et al., 2009). When *ρ* = 0.30 and power = 0.80, the minimum sample size required is 84 people. In this study, 117 data samples were randomly selected from the Study 3 samples for the analysis of effect size association validity.

To compute the test–retest reliability, we asked 64 participants whose voluntarily participated the retested study among the 671 participants in Study 3 to complete the survey twice, with a two-week interval between the two surveys.

#### Materials

##### Chinese ethnic trust scale

The 24-items intergroup ethnic trust scale and the 24-items interpersonal ethnic trust scale developed in the Study 2 were administered to the participants (see the Questionnaire in Supplementary material). Items were rated on a 7-point Likert scale (1 = “strongly disagreed,” 4 = “uncertain,” 7 = “strongly agreed”). Higher scares indicated greater levels of ethnic trust.

##### Interpersonal trust scale

The 10-items revised Interpersonal Trust Scale (ITS) was used to measure the extent to which participants’ estimation of the reliability of others’ behavior, commitments, or statements, including two dimensions of trust in social phenomena (6 items) and commitment behavior (4 items) (Ding and Peng, 2020). The ITS items were rated on a 5-point Likert scale ranging from 1 (strongly disagree) to 5 (strongly agree), with higher scores indicating higher feelings of trust. The ITS has a good reliability and validity. In this study, the Cronbach’s *α* coefficients for the entire scale and the two dimensions were 0.855, 0.852, and 0.769, respectively.

##### Ethnic unity awareness scale

The 22-items Ethnic Unity Awareness Scale (EUAS) was used to measure the extent to which participants’ ethnic unity consciousness among adolescents and adults in various regions of China, including three dimensions of ethnic intention (9 items), ethnic cognition (8 items), and ethnic emotion (5 items) (Chen and Fan, 2021). The EUAS items were rated on a 5-point Likert scale ranging from 1 (strongly disagree) to 5 (strongly agree), with higher scores indicating higher consciousness of ethnic unity. The EUAS has a good reliability and validity. In this study, the Cronbach’s α coefficients for the entire scale and the three dimensions were 0.939, 0.862, 0.900, and 0.875, respectively.

#### Procedure

A link to the survey was posted to the participants. An information sheet was presented first, followed by an informed consent form. Before the survey began, participants were required to provide a self-generated unique identification code. This was used to match participants to the second repeat survey to assess the test–retest reliability of the Chinese Ethnic trust scale. Six hundred and sixty-seven participants then completed the Chinese Ethnic trust scale and 117 participants of 667 completed each of the three questionnaires, including the Chinese Ethnic trust scale, Interpersonal Trust Scale (Ding and Peng, 2020) and Ethnic Unity Awareness Scale (Chen and Fan, 2021). At the beginning of each questionnaire, a brief “instruction” described how to complete each questionnaire.

At the end of this survey, participants could decide whether they wanted to participate in a follow-up evaluation to assess the test–retest reliability of the Chinese Ethnic trust scale. If they wished to do so, they provided their email address so they could be contacted with a link to the second completion of the Chinese Ethnic trust scale. Two weeks after completing the Chinese Ethnic trust scale for the first time, participants were emailed a link to complete the Chinese Ethnic trust scale again, also through Tencent Questionnaire Platform. An information sheet was provided, and participants gave their consent. Participants entered their unique identification code that they had included in the first survey. Then participants completed the Chinese Ethnic trust scale for the second time.

The participants filled in the questionnaire through the online website. AMOS 24.0 software was used to analyze the two-factor structure of the ethnic interpersonal/intergroup-trust scale obtained from Study 2. Based on the common evaluation index requirements in psychometric questionnaire development, the selected indicators include χ^2^/*df*, root mean square error of approximation (RMSEA), standardized RMR (SRMR), Comparative Fit Index (CFI), Tucker-Lewis Index (TLI) and Incremental fit Index (IFI).

Since the two factors of ethnic interpersonal/intergroup trust may also constitute a single interpersonal trust dimension, we conducted confirmatory testing and comparison between the single-dimensional model and the two-dimensional model of interpersonal/intergroup trust as competitive models simultaneously. The maximum likelihood method was chosen for the model parameter estimation to explore the relationship between items and latent variables.

Furthermore, due to the possibility of consolidating inter-ethnic trust at both the interpersonal and intergroup levels into a single dimension of ethnic trust, or alternatively, dividing it into four dimensions: interpersonal universal ethnic trust, interpersonal particular ethnic trust, intergroup universal trust, and intergroup particular trust. Therefore, we established three theoretical models and four competing models, including the two-factor second-order model of interpersonal and intergroup trust orientation, the universal and particular trust two-factor model of interperson/intergroup orientation, the four-factor first-order model (interpersonal universal trust, interpersonal particular trust, intergroup universal trust, and intergroup particular trust), the single-dimensional model (ethnic trust), and the single-dimensional model of interperson/intergroup orientation trust. The maximum likelihood method was chosen for the model parameter estimation to explore the relationship between items and latent variables. Additional, Cross-validity analysis and Akaike information criterion were used to further compare model superiority or inferiority, with smaller ΔAIC and ΔECVI values indicating a better model fit (Liu et al., 2007).

### Result

#### Confirmatory factor analysis of the two-order model on the interpersonal trust

**Figure 2:**
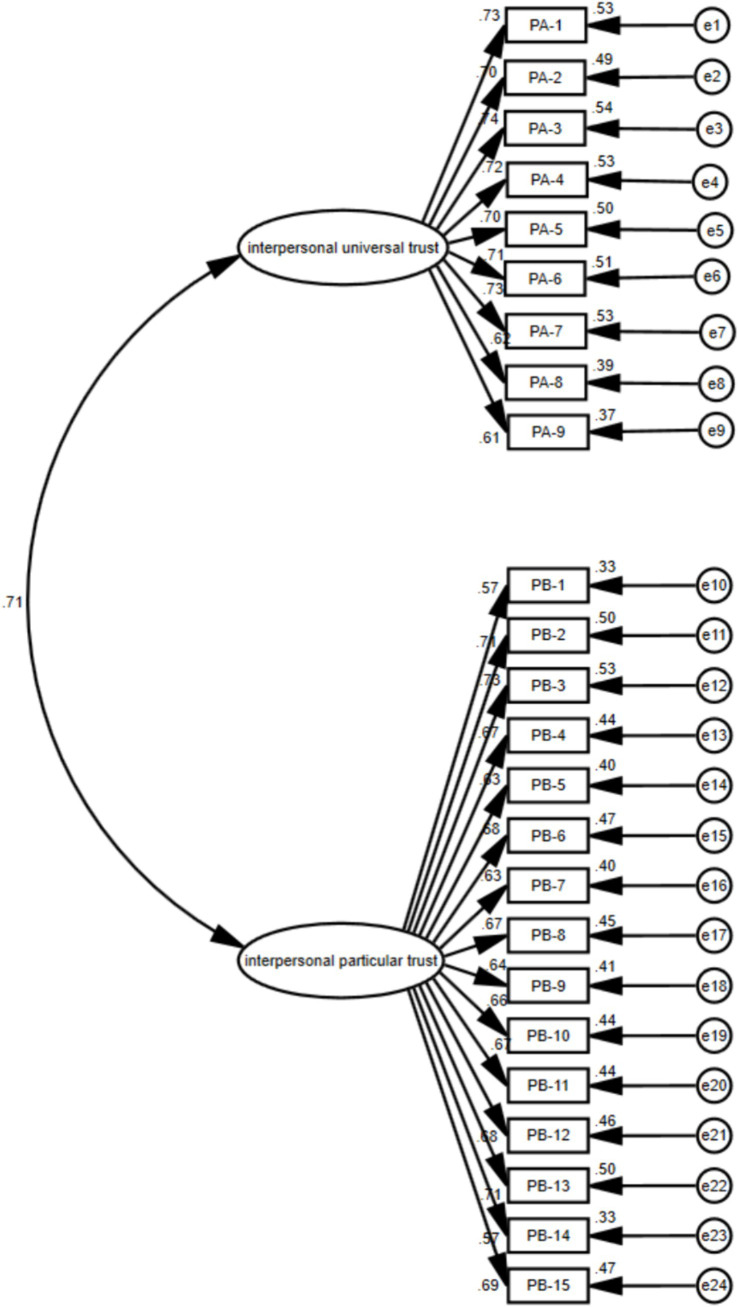
The two-factor model of interpersonal-orientation ethnic trust. PA1-9 represents the items of the dimension of interpersonal universal trust; PB1-15 represents the items of the dimension of interpersonal particular trust.

Based on the common evaluation index requirements in psychometric questionnaire development, the value of χ^2^/*df* in ranges of 1 to 5 as an indicator of a good fit. CFI, TLI and IFI’s recommended values should be greater than 0.90 or approaching 0.9, RMSEA and SRMR are less than 0.08 for a good model fit (Wu, 2010). The results of the two-factor structure of the goodness-of-fit were as follows: χ^2^/*df* < 5; RMSEA and SRMR were lower than 0.05; CFI, TLI and IFI were greater than 0.90. The results indicated that the model was within the acceptable fit indexes (Table 7 and Figure 2).

**Table 7:** Fit indices of the two-factor interpersonal trust model (*n* = 671).

Fit indices	χ2/*df*	RMSEA [90% CI]	SRMR	CFI	TLI	IFI
Two-order model	4.29	0.07 [0.066–0.074]	0.052	0.90	0.88	0.90
Competition model-single dimension model	9.41	0.112 [0.108–0.116]	0.156	0.73	0.70	0.73

#### Confirmatory factor analysis of the two-order model on the intergroup trust

**Figure 3:**
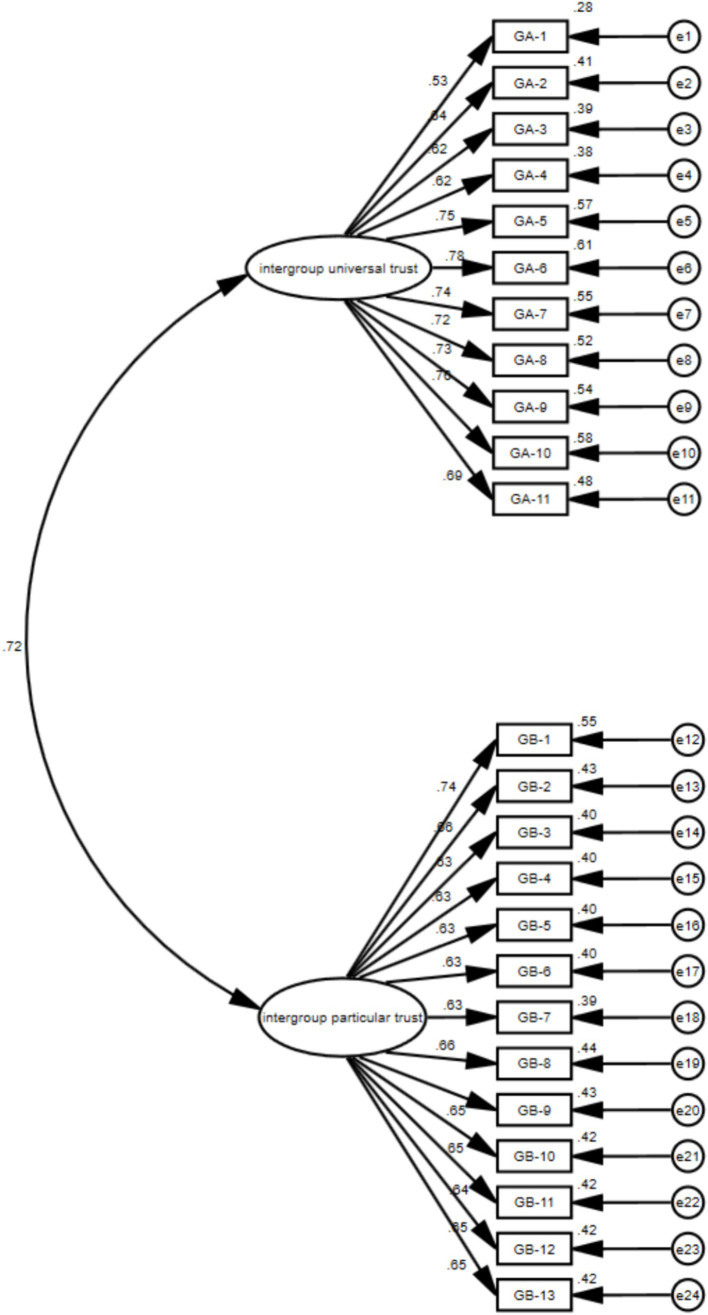
The two-factor model of intergroup-orientation ethnic trust. GA1-11 represents the items of the dimension of intergroup universal trust; GB1-13 represents the items of the dimension of intergroup particular trust.

The results of the two-factor structure of the goodness-of-fit were as follows: χ2/*df* < 5; RMSEA and SRMR were lower than 0.05; CFI, TLI and IFI were greater than 0.90. The results indicated that the model was within the acceptable fit indexes (Table 8 and Figure 3).

**Table 8:** Fit indices of the two-factor intergroup trust model (*n* = 671).

Fit indices	χ2/df	RMSEA [90% CI]	SRMR	CFI	TLI	IFI
Two-order model	3.19	0.057 [0.053–0.062]	0.0496	0.93	0.92	0.93
Competition model-single dimension model	6.63	0.092 [0.088–0.096]	0.0755	0.81	0.79	0.81

Furthermore, the comparative results of the three theoretical models and four competing models indicate that except for the poor fit of the single-dimensional model, it is not possible to judge the superiority or inferiority of these models solely based on the indicators of χ^2^/*df*, RMSEA, SRMR, CFI, TLI, and IFI, which indirectly indicates that the two sub-scales can be combined to form a ethnic trust total scale (as shown in Table 9). Cross-validity analysis and Akaike information criterion were used to further compare model superiority or inferiority, with smaller ΔAIC and ΔECVI values indicating a better model fit. As shown in Table 10, the ΔAIC and ΔECVI values of the two-factor interpersonal model and the two-factor intergroup model are smaller than those of other models, which is consistent with our theoretical hypothesis, indicating that ethnic trust can be divided into interpersonal trust and intergroup trust components. The models of the two sub-scales each containing 2 components are reasonably set.

**Table 9:** Index of goodness of fit for the Chinese ethnic trust scale (*n* = 671).

Fitting indicators	χ^2^/*df*	RMSEA	SRMR	CFI	TLI	IFI
The two-order four-factors model of ethnic trust	3.06	0.06	0.06	0.87	0.86	0.87
The four-factor first-order model	2.97	0.05	0.06	0.87	0.86	0.87
The single-dimensional model of ethnic trust	7.16	0.10	0.11	0.60	0.58	0.60
The two-order model of interpersonal trust	3.77	0.06	0.05	0.90	0.91	0.91
The two-order model of intergroup trust	3.18	0.06	0.05	0.93	0.92	0.93
The single-dimensional model of interpersonal trust	9.41	0.11	0.16	0.73	0.70	0.73
The single-dimensional model of intergroup trust	6.63	0.09	0.076	0.81	0.80	0.81

**Table 10:** Comparison of competition model superiority and inferiority indicators (*n* = 671).

Fitting indicators	ECVI	ECVI saturated/ECVI independent	ΔECVI	AIC	AIC saturated/AIC independent	ΔAIC
The two-order four-factors model of ethnic trust	5.21	3.51/26.30	1.70	3428.67	2352/21389.81	1076.67
The four-factor first-order model	5.07	3.51/26.30	1.56	3393.99	2352/17621.53	1041.99
The single-dimensional model of ethnic trust	11.82	3.51/26.30	8.31	7921.33	2352/17621.53	5569.33
The two-order model of interpersonal trust	1.55	0.90/12.17	0.65	1041.35	600/8155.50	441.35
The two-order model of intergroup trust	1.34	0.90/11.60	0.44	896.58	600/7773.38	296.58

#### Construct validity

The construct validity includes both convergent validity and discriminant validity, measured by the correlation coefficients between the total scores of the sub-scale and the total score, between the total score of the sub-scale and the factor, between factors, the combination reliability of the two dimensions of each sub-scale, and the average variance extracted. The results show that the correlation coefficient between the sub-scale and the total score is between 0.81 and 0.90 (*p* < 0.001), and the correlation coefficient between the two sub-scales is 0.47 (*p* < 0.001). The correlation coefficient between the sub-scale and its corresponding factor is between 0.90 and 0.91 (*p* < 0.001), and the correlation coefficient between factors is between 0.63 and 0.65 (*p* < 0.001). The correlation between the total score of the scale and the sub-scale is higher than the correlation between the sub-scales, and the correlation between the sub-scale and the factor is higher than the correlation between factors, indicating good convergent validity, all measuring the same trait. The moderate correlations between sub-scales and factors indicate that the trait can be measured in different dimensions, showing good discriminant validity.

In addition, the combination reliability CR (>0.80), standardized factor loadings (0.53–0.78, all greater than 0.50), and average variance extracted AVE (0.42–0.49, all greater than 0.40) all reached acceptable levels, further indicating good convergent validity of the scale. The square root of the average extracted variance of each latent variable (0.65–0.70) is greater than the correlation coefficients between that latent variable and other latent variables, indicating good discriminant validity (Wu, 2010).

#### Criterion-related validity

The criterion-related validity was examined by calculating the correlations between the Chinese ethnic trust scale’ scores and the Interpersonal Trust Scale and Ethnic Unity Awareness Scale. The correlation results between the ethnic trust scales of interpersonal orientation and intergroup orientation with the above scales are shown in Table 11. The interpersonal orientation ethnic trust and intergroup orientation ethnic trust are positively correlated with each dimension of the Ethnic Unity Awareness Scale, indicating good criterion-related validity of the above scales and measuring the same trait. The interpersonal orientation ethnic trust is positively correlated with each dimension of the Interpersonal Trust Scale, while the intergroup orientation ethnic trust is not significantly related to each dimension of the Interpersonal Trust Scale, further demonstrating the good discriminant validity of the two subscales of the ethnic trust scale.

**Table 11:** Correlation between the ethnic trust scale, interpersonal trust scale, and national unity consciousness scale in various dimensions.

	Social phenomenon trust	Commitment leads to trust	National Intention	National cognition	National emotion
Interpersonal oriented ethnic trust	0.216^**^	0.224^*^	0.454^***^	0.319^***^	0.249^***^
Intergroup oriented ethnic trust	−0.023	−0.066	0.767^***^	0.750^***^	0.635^***^

^*^p < 0.05, ^**^p < 0.01, ^***^p < 0.001.

#### Internal consistency reliability

The Cronbach’s a of the total scale of ethnic trust, interpersonal-oriented ethnic trust, intergroup-oriented ethnic trust, interpersonal-oriented universal ethnic trust, interpersonal-oriented particular ethnic trust, intergroup-oriented universal ethnic trust, intergroup-oriented particular ethnic trust were between 0.863 and 0.955, all higher than 0.85, indicating high internal consistency reliability (Wu, 2010).

#### Test–retest reliability

Test–retest reliability was analyzed by correlating completions of the ethnic trust at Time 1 with those at Time 2 (2 weeks later) by 64 participants whose voluntarily participated in the follow-up study were retested among the 671 participants in Study 3. The results showed that the total ethnic trust, interpersonal-orientation ethnic trust, intergroup-orientation ethnic trust, interpersonal-orientation particular ethnic trust, interpersonal-orientation universal ethnic trust, intergroup-orientation universal ethnic trust, and intergroup-orientation particular ethnic trust were 0.930, 0.834, 0.798, 0.710, 0.838, 0.856, and 0.735 respectively, all greater than 0.70, indicating that the test–retest reliability of the ethnic trust is good (Wu, 2010).

## Discussion

This article constructs the concept structure of Chinese ethnic trust and develops corresponding measurement tools by combining theoretical and data-oriented approaches, further verifying the validity of the concept structure.

### Concept structure of the ethnic trust

Through free association and in-depth interviewing method, this article proposes the concept structure of trust among the Chinese people, namely interpersonal universal trust, interpersonal particular trust, intergroup universal trust, and intergroup particular trust.

Interpersonal universal trust is the result of people making rational judgments about others’ ability, benevolence, and integrity based on established facts, which basically correspond to the trust integration model. This theory believes that the trustee’s benevolent, integrity, and ability will determine the trust perception results of the trustor (Colquitt et al., 2007; Wang et al., 2020). Based on the content of social judgment, people typically perceive others’ personal traits from three dimensions: competence, warmth, and morality (Ellemers et al., 2017). Therefore, the basic dimensions of social judgment can serve as the fundamental content for interpersonal trust judgments, where the dimensions of competence and ability, warmth and benevolence, and morality and integrity correspond, respectively. Benevolence refers to the positive trait of being able to exclude self-interest motivations, being oriented towards mutual interests, and voluntarily helping others, including friendly, warm, and caring behaviors (Mayer et al., 1995). These traits correspond to the dimension of warmth in social judgment (Ellemers et al., 2017), which are important standards for measuring the level of trust in the trustee. In Chinese culture, benevolent has been a friendly sign since ancient times, similar to benevolence and compassion (Wang et al., 2020; Li et al., 2021). This trait also exhibits cross-cultural consistency. Based on the moral foundation theory, humans are born with a “draft” of the moral mind, that is, moral foundations, and it is believed that people are “born to be righteous” (Haidt and Graham, 2007). The moral foundation of “care” is not significantly different in importance across various cultural groups (Haidt and Joseph, 2004). Integrity trait reflects people’s perception judgment of others’ reliability and consistency in words and actions (Mayer et al., 1995). These traits correspond to the dimension of morality in social judgment (Ellemers et al., 2017), which are consistent with the high-frequency words in this study such as “honesty,” “reliability,” and “steadfastness,” and interview examples such as “I judge whether they are reliable based on my personal feelings, not so much related to ethnicity.” Ability trait refers to the skills, competence, and characteristics that individuals possess, enabling them to have influence in particular areas (Mayer et al., 1995). Trust involves risk, and controlling risk requires certain conditions to be met. In real life, when people perceive that the other party has high ability, they are more inclined to trust them (Cook and Wall, 1980; Deutsch, 1960; Sitkin and Roth, 1993). Ability trust refers to one party’s ability to complete a certain action according to the other party’s requirements and expectations. People’s perception of others’ high capability traits can control the risks associated with the inability to complete tasks (Ellemers et al., 2017). As interviewees mentioned “No matter which ethnic group a person belongs to, if he has the capability in a certain field, I am willing to trust him for that reason and willing to cooperate with him in that field.” and “I trust those who are responsible.” This is consistent with previous research findings that there is a significant positive correlation between investors’ trust in entrepreneurs’ personal capabilities and the scale of investment (Yu and Pan, 2008) and is also consistent with high-frequency words in this study such as “confidence.” To emphasize the connection between the trust integration model and social judgment, we named the three dimensions of universal interpersonal trust to the ability-competence, benevolence-warmth, and integrity-morality.

Interpersonal particular trust includes interpersonal emotional reciprocity, close relationships and mutual assistance, and tendency to express emotions, representing positive interactive relationships among members of various ethnic groups, with connotations of both domestic and western consistency and unique characteristics of China. On the one hand, behaviors such as “mutual trust,” “mutual assistance,” “truthfulness,” are similar to friendly interactions among members of different races abroad, such as telling the truth and providing support and help, as well as daily life interactions among members of different ethnic groups domestically, such as taking care of children, borrowing phones, holding keys, lending money, etc. On the other hand, the positive interactive relationships among members of various ethnic groups in China are not only reflected in times of prosperity with “common progress, common development, shared wealth, mutual dependence,” but also in times of adversity with “sharing honor and disgrace, sharing hardships, mutual support, and solidarity,” which continues the tradition of collective welfare in Chinese collectivism and its self-concept is mainly defined based on social embeddedness and interdependence with others, including members of the in-group (Brewer and Chen, 2007). This is fundamentally different from the rational calculations and exchange contracts of interests and losses in Western individualism (Ren, 2021; Yang, 1991).

Intergroup universal trust and intergroup particular trust are the two dimensions of intergroup-oriented ethnic trust. The former includes the perception of fairness and justice, common societal values, and peaceful association, which have more consistency with domestic literature and have Chinese characteristics. Particularally, the content of “perception of fairness and justice” such as equal treatment of all ethnic groups, equal social rights, and responsibilities are consistent with the idea of “equal mutual trust among all ethnic groups, achieving fair and reasonable distribution of rights among ethnic groups, and equal enjoyment of ethnic interests” mentioned by Zhu and Zhou (2014). This may be due to the Marxist national view held by China, which believes that the core of solving national issues is to achieve ethnic equality, where all ethnic groups are equal in rights and privileges (Li and Yan, 2013). Under this normative concept, 56 ethnic groups in China place more emphasis on the equality of relationships. Similarly, the main content of “common societal values” includes traditional Chinese cultural values, core values of modernity, and the great rejuvenation goal of the Chinese nation in the future, reflecting a collective level of value consensus and the domain of moralistic trust (Yuan, 2015). Yuan (2015) used Sichuan Province as an example and found that ethnic trust is formed in people’s ethical practices of interacting with each other, based on expectations of good virtues and good things. This also reflects the moralistic trust in value consensus similar to the “common societal values.” While western researchers have mentioned that common values are an important dimension of trust, it is important to consider that different civilizations, countries, and regions have different natural conditions and developmental histories, which often lead to the formation of distinct and unique cultural values (Nooteboom, 2004; Zerfu et al., 2009). For example, by comparing the moral foundations across different cultural circles, the results show that there is no difference between Eastern and Western cultural groups in terms of moral foundations such as care, justice, honesty, and authority. However, the former rates the moral foundations of loyalty and sanctity higher than the latter (Haidt and Joseph, 2004). Therefore, the content of “common socialist values” in this study can also be considered noteworthy. The variation in social values among different countries may be associated with the cultural tightness–looseness states. The cultural tightness–looseness theory defines the tightness or looseness of a group’s culture as the collective perceptions among its members about the strength of the group’s internal social norms and the degree of consensus on these norms (Gelfand, 2018). The significant positive correlation between China’s collectivist values and cultural tightness indicates that collectivist values might promote a more cohesive and regulated cultural environment, which helps in forming and sustaining social trust (Chua et al., 2019). This trust profoundly influences people’s values, worldviews, environment, and cognition, thus fostering the development of common values with distinctive Chinese characteristics. The component of peaceful association in intergroup universal trust exhibits cross-cultural consistency. Domestic peaceful interactions should ideally present a “state of no suspicion or doubt, where different ethnic groups or races peacefully coexist” as previous researchers have suggested (Li and Liang, 2020; Wang and Wu, 2018; Wang, 2017). Western researchers developed an inter-group trust scale based on the historical background of Cyprus (Psaltis, 2011). Through three questions, they measured the trust between Greek Cypriots and Turkish Cypriots, with one question asking, “When Greek/Turkish Cypriots say they want peace, I trust them,” which illustrates that “peaceful interactions” is an important component of inter-group trust.

The particular trust between groups includes ethnic positive emotions, economic interconnection and mutual assistance, and cultural exchange and symbiosis. In terms of ethnic positive emotions, in addition to intimacy and security (Cheng, 2020; Li and Wu, 2014), this study also found content related to dependency and cohesion, such as “brothers and sisters,” “cohesion,” “pomegranate seeds,” “big family,” “seeking common ground while preserving differences,” “ethnic intermarriage,” “a close-knit family,” etc., which are highly distinctive Chinese characteristics and further enrich the emotional content of ethnic trust domestically. Cultural exchange and symbiosis include various contents, which are in line with the views of numerous domestic and western researchers, such as recognizing and respecting various ethnic cultures (Yuan, 2015), cultural interactions (Zhu and Zhou, 2014), ethnic intermarriage (Zhu and Zhou, 2014), mutual participation in ethnic activities (Yuan, 2015; Ingelaere, 2016), and so on. The components of economic interconnection and mutual assistance were only found in qualitative research results, but this component is not a new discovery. The classic game theoretical framework of ethnic trust in the past quantified trust from an economic perspective (Gong et al., 2021; Elena et al., 2015; Kayaoglu, 2017; Navarro-Carrillo et al., 2018), which also indirectly reflects that economic interconnection and mutual assistance are part of the connotation of ethnic trust.

In conclusion, the conceptual structure of ethnic trust obtained in this study not only has the characteristics of self-categorization between interpersonal and intergroup levels but also includes the relational dimensions between universal and particular trust. The self categorization theory suggests that people not only perceive unique individual identities, but also acquire various social identities based on their membership in different social groups (Tajfel, 1978; Turner and West, 2012). Human behavior can be as a continuum, with one end being interpersonal behavior, and people acting as individuals; On the other hand, there is intergroup behavior, where people act as members of a group, involving differences in behavior between different groups (Tajfel, 1978). Consequently, when ethnic identities remain dormant and individuals from various ethnic backgrounds engage with one another on a personal level, the trust that emerges is interpersonal in nature. It is shaped by direct interactions that reflect the values and attitudes of the individuals involved, with ethnic identity playing a minimal role in this dynamic. However, once ethnic identities are brought to the forefront, individuals identify themselves as part of a specific ethnic group rather than as isolated individuals. In such cases, when interactions occur between members of different ethnic groups, the resulting trust is intergroup trust, reflecting a collective identity and the dynamics of group-to-group relationships. The relational dimension reflects the unique “differential order pattern” of Chinese relationship modes. The “differential order pattern” was proposed by Fei (1980), who believed that Chinese relationships are not simply linear or hierarchical structures, but rather a ripple-like structure that spreads out from the individual as the center. This relationship pattern emphasizes the individual’s central position in the social network, as well as their influence and the radiating nature of their social circle. The results of this study show that the trust relationships among Chinese ethnic groups exhibit a graded pattern of trust based on emotions, which includes both the special trust among ethnic groups based on familiarity in terms of region, kinship, and culture, and the universal trust among a broader range of ethnic groups in unfamiliar environments.

The results expand the previous single-dimensional structure of domestic and western ethnic trust concepts (interpersonal or intergroup trust, particular or universal trust) to a dual-dimensional structure, demonstrating the multi-level relationship characteristics between interpersonal and intergroup relations of various ethnic groups in China, and conforming to the “skewed trust pattern with emotions at the center” in the Chinese cultural context. This represents a development and advancement of the conceptual structure of ethnic trust.

### Reliability and validity of the ethnic trust scale

The compilation of the Ethnic Trust Scale follows the standards of psychometrics, distinguishing itself from the interpersonal trust scale (Rotter, 1967) by including not only benevolent content but also integrating integrity and perceived ability content. In addition, the Ethnic Trust Scale highlights the characteristics of the Chinese ethnic groups. For example, “When facing external threats, Chinese ethnic groups can unite” better reflects the strong Chinese indigenous characteristics. Exploratory and confirmatory factor analyses empirically substantiate the rationality of the conceptual structure of ethnic trust. Validity and reliability tests show that both interpersonal/intergroup orientations demonstrate good internal consistency and cross-temporal stability (such as Cronbach’s alpha coefficient > 0.70, CR > 0.80, test–retest reliability >0.70). One of the criterion tools, the National Unity Consciousness Scale, is used to measure the unity relationship between various Chinese ethnic groups, encompassing both interpersonal aspects (e.g., “I am willing to be friends with members of other ethnic groups”) and intergroup aspects (e.g., “The common goal of the 56 ethnic groups is to realize the great rejuvenation of the Chinese nation and the Chinese dream”). It should be positively correlated with both interpersonal/intergroup orientations of ethnic trust. The second criterion tool, the revised Chinese version of the Interpersonal Trust Scale, is used to measure trust relationships between individuals. It should be positively correlated with interpersonal orientations of ethnic trust, and the data results confirm these hypotheses, indicating that this scale has good criterion-related validity.

Based on the above, the Ethnic Trust Scale meets the standards of psychometrics and provides an effective measurement tool for understanding the level of trust among Chinese ethnic groups as well as for future empirical research. However, this study can still be improved. For example, although it involves many ethnic groups and has a wide coverage of participants, the overall number of participants is relatively small. Therefore, future research could be widely conducted in various regions and among different occupational groups, expanding the scope of sample testing to gain a comprehensive understanding of the current level of trust among Chinese ethnic groups.

## Conclusion

This study yielded two conclusions through three research approaches. First, the structure of ethnic trust contains two dimensions (interpersonal-oriented ethnic trust, intergroup-oriented ethnic trust) and four factors named interpersonal universal trust, interpersonal particular trust, intergroup universal trust, and intergroup particular trust. Second, the ethnic trust scale consists of two subscales: the interpersonal-oriented ethnic trust scale and the intergroup-oriented ethnic trust scale. Each subscale includes two factors of particular trust and universal trust, with 24 items each, totaling 48 items. The questionnaire has good reliability and validity and can be used as an effective tool to measure the degree of trust relationships among different ethnic groups in China.

## Data Availability

The raw data supporting the conclusions of this article will be made available by the authors, without undue reservation.
