# Supplementary material for: Development and validation of the ethnic trust scale in China
Source: Front Psychol. 2024 Oct 14;15:1394819. doi: 10.3389/fpsyg.2024.1394819 (PMC11514384; doi:10.3389/fpsyg.2024.1394819)
Supplement: Supplementary file 1 [file Table_1.docx]

| **Description of the Interpersonal Ethnic Trust Scale** | strongly disagree | relatively disagreed | uncertain | relatively agreed | strongly agree |
| --- | --- | --- | --- | --- | --- |
| 1. No matter which ethnicity, the vast majority of people are friendly. |  |  |  |  |  |
| 1. I can usually feel the benevolent naturally expressed by people of all ethnic groups. |  |  |  |  |  |
| 1. I think that the majority of people from different ethnic groups are quite diligent in their work. |  |  |  |  |  |
| 1. I think that honesty is the best quality for people of any ethnicity. |  |  |  |  |  |
| 1. Most people of all ethnic groups are trustworthy in keeping their promises. |  |  |  |  |  |
| 1. I believe every ethnic group has excellent individuals in a certain professional field. |  |  |  |  |  |
| 1. I believe that the majority of people in all ethnic groups are capable of doing their own work well. |  |  |  |  |  |
| 1. I am willing to cooperate with capable people regardless of their ethnicity. |  |  |  |  |  |
| 1. When others have strong professional skills, I do not mind their ethnic identity information. |  |  |  |  |  |
| 1. If I entrust important matters to cross-ethnic relatives, friends, neighbors, or other acquaintances, I do not need to explain and closely supervise repeatedly. |  |  |  |  |  |
| 1. I will be very relieved to hand things over to a cross-ethnic acquaintance for handling. |  |  |  |  |  |
| 1. When facing difficulties and setbacks, I can receive care, understanding, or encouragement from friends of different ethnic groups. |  |  |  |  |  |
| 1. I often confide my troubles with my close friends from different ethnic groups. |  |  |  |  |  |
| 1. I can confide secrets to relatives, friends, neighbors, or colleagues of different ethnicities. |  |  |  |  |  |
| 1. Besides friends from my own ethnic group, I also want to share happy with friends of different ethnicities as soon as possible (such as good news in life). |  |  |  |  |  |
| 1. In front of trusted cross-ethnic members, I will not hide my true thoughts. |  |  |  |  |  |
| 1. I believe that acquaintances such as cross-ethnic relatives, friends, neighbors, or colleagues will not joke with me maliciously. |  |  |  |  |  |
| 1. I and cross-ethnic relatives, friends, neighbors, or colleagues and other acquaintances, will not misinterpret each other’s intentions when joking. |  |  |  |  |  |
| 1. Sometimes when I communicate with acquaintances (such as relatives, friends, neighbors or colleagues) from different ethnic backgrounds, I naturally use a joking tone to express my thoughts and feelings. |  |  |  |  |  |
| 1. Cross-ethnic relatives, friends, neighbors, colleagues, and other acquaintances often invite me to share food. |  |  |  |  |  |
| 1. In daily life, I am willing to accompany acquaintances from different ethnic groups. |  |  |  |  |  |
| 1. When I need help, I believe that relatives, friends, neighbors, or colleagues from different ethnic groups will do their best to help me. |  |  |  |  |  |
| 1. When I ask for help from friends of different ethnic backgrounds, I am not worried about causing trouble for them. |  |  |  |  |  |
| 1. When help is needed, I will actively seek help from other trusted members of different ethnic backgrounds. |  |  |  |  |  |

| **人际取向民族信任量表的描述** | 完全  不符合 | 比较  不符合 | 不确定 | 比较  符合 | 完全  符合 |
| --- | --- | --- | --- | --- | --- |
| 1. 无论哪个民族，绝大多数的人都是友善的。 |  |  |  |  |  |
| 1. 我通常能感受到各民族自然流露出来的善意。 |  |  |  |  |  |
| 1. 我认为各民族的大多数人做事都比较踏实。 |  |  |  |  |  |
| 1. 我认为无论哪个民族的人，诚信都是最好的品质。 |  |  |  |  |  |
| 1. 各民族中的大多数人在遵守诺言方面是值得信任的。 |  |  |  |  |  |
| 1. 我相信每个民族都有在某一专业领域表现出色的人。 |  |  |  |  |  |
| 1. 我相信各民族中的大多数人都能做好自己的工作。 |  |  |  |  |  |
| 1. 我愿意和能力强的人合作，与他所属民族身份无关。 |  |  |  |  |  |
| 1. 当对方的专业能力较强时，我不会在意他的民族身份信息。 |  |  |  |  |  |
| 1. 如果将重要事情交给跨民族的亲戚、朋友、邻居或同事等熟人处理，我不需要反复交代和密切监督。 |  |  |  |  |  |
| 1. 我会很放心把事情交给跨民族熟人去处理。 |  |  |  |  |  |
| 1. 在面临困境与挫折时, 我能得到来自跨民族亲友的关心、理解或鼓励。 |  |  |  |  |  |
| 1. 我常和知心的跨民族成员相互倾诉烦恼。 |  |  |  |  |  |
| 1. 我能向跨民族的亲戚、朋友、邻居或同事等熟人吐露秘密。 |  |  |  |  |  |
| 1. 除本民族朋友外，我也想第一时间和跨民族朋友分享喜悦（如生活的好消息等）。 |  |  |  |  |  |
| 1. 在知心的跨民族成员面前，我不会隐瞒自己的真实想法。 |  |  |  |  |  |
| 1. 我相信跨民族的亲戚、朋友、邻居或同事等熟人在和我开玩笑时并无恶意。 |  |  |  |  |  |
| 1. 我和跨民族的亲戚、朋友、邻居或同事等熟人在开玩笑时，不会曲解彼此的意思。 |  |  |  |  |  |
| 1. 我有时候和跨民族的亲戚、朋友、邻居或同事等熟人交流时，会很自然地使用开玩笑的口吻来表达自己的想法和感受。 |  |  |  |  |  |
| 1. 跨民族的亲戚、朋友、邻居或同事等熟人经常邀请我共享美食。 |  |  |  |  |  |
| 1. 在日常生活中，我愿意和跨民族熟人相伴而行。 |  |  |  |  |  |
| 1. 在我需要帮助时，我相信跨民族的亲戚、朋友、邻居或同事等熟人会尽其所能帮助我。 |  |  |  |  |  |
| 1. 当我向跨民族亲友求助时，我不担心会给对方制造麻烦。 |  |  |  |  |  |
| 1. 当需要帮助时，我会主动向可信赖的其他民族成员求助。 |  |  |  |  |  |

| **Description of the Intergroup Trust Scale** | strongly disagree | relatively disagreed | uncertain | relatively agreed | strongly agree |
| --- | --- | --- | --- | --- | --- |
| 1. My attitude towards my own ethnic and other ethnic is the same. |  |  |  |  |  |
| 1. All ethnic group have the right to equal participation in the political life of our country. |  |  |  |  |  |
| 1. Every ethnic group will consciously safeguard the unity of the motherland and oppose secession. |  |  |  |  |  |
| 1. I am not afraid to reveal or show my ethnicity in public settings. |  |  |  |  |  |
| 1. I believe that the 56 ethnic groups in China can live together harmoniously. |  |  |  |  |  |
| 1. The core socialist values are an important bond for maintaining trust between the 56 ethnic groups in the country. |  |  |  |  |  |
| 1. The core socialist values are common spiritual norms shared by all ethnic groups. |  |  |  |  |  |
| 1. The Chinese cultural concept of “Harmony under Heaven” binds together various ethnic groups as one. |  |  |  |  |  |
| 1. Integrity is moral standards that all ethnic groups need to abide by. |  |  |  |  |  |
| 1. The great rejuvenation of the Chinese nation is a common value guidance for all ethnic groups. |  |  |  |  |  |
| 1. When facing external threats, the 56 ethnic groups in China can unite. |  |  |  |  |  |
| 1. When interacting with familiar ethnic groups, I can’t help but see us as one entity. |  |  |  |  |  |
| 1. All ethnic groups work together and support each other in economic development. |  |  |  |  |  |
| 1. I believe that familiar cross-ethnic groups will not intentionally violate my ethnic taboos. |  |  |  |  |  |
| 1. Compared to unfamiliar groups, I can better understand the unique cultures of other familiar ethnicities. |  |  |  |  |  |
| 1. When other ethnic groups use their unique languages, I believe it is simply because they are more proficient in their own languages. |  |  |  |  |  |
| 1. I like to share books, videos, and other materials related to my own ethnic culture with other ethnic groups that I am familiar with. |  |  |  |  |  |
| 1. I am very willing to listen to people from other ethnic groups share the unique aspects of their ethnicities (such as cultural customs, etc.). |  |  |  |  |  |
| 1. I am willing to deeply communicate with other ethnic groups about the cultures customs of our respective ethnicities. |  |  |  |  |  |
| 1. I will consider the different customs of my own ethnic group and other ethnic groups as distinctive cultural features of each other. |  |  |  |  |  |
| 1. I am willing to actively learn about the unique cultures of other ethnic groups. |  |  |  |  |  |
| 1. Compared to unfamiliar groups, I prefer to embrace familiar cross-cultural customs. |  |  |  |  |  |
| 1. I am willing to trust the familiar ethnic group because our cultures blend more. |  |  |  |  |  |
| 1. universally, people will not reject interethnic marriage if they have a high level of trust in other ethnic groups. |  |  |  |  |  |

| **群际取向民族信任量表的描述** | 完全  不符合 | 比较  不符合 | 不确定 | 比较  符合 | 完全  符合 |
| --- | --- | --- | --- | --- | --- |
| 1. 我对本民族和他民族的态度是一致的。 |  |  |  |  |  |
| 1. 我们各民族拥有平等的参与国家政治生活的权利。 |  |  |  |  |  |
| 1. 我们各民族都会自觉维护祖国统一，反对分裂。 |  |  |  |  |  |
| 1. 我不怕在公众场合透露或者表现我的民族身份。 |  |  |  |  |  |
| 1. 我认为中国各民族可以和谐共处。 |  |  |  |  |  |
| 1. 社会主义核心价值观是国内56个民族间维系信任关系的重要纽带。 |  |  |  |  |  |
| 1. 社会主义核心价值观是各民族共有的精神规范。 |  |  |  |  |  |
| 1. 中国“天下大同”的文化将各民族拧成一股绳。 |  |  |  |  |  |
| 1. “诚实守信”是各民族都需要遵守的道德规范。 |  |  |  |  |  |
| 1. 实现中华民族伟大复兴是各民族共同的价值引领。 |  |  |  |  |  |
| 1. 当有外来威胁时，中国各民族能团结一致。 |  |  |  |  |  |
| 1. 与熟悉的其他民族群体交往时，我会不由自主地将我们视为一体。 |  |  |  |  |  |
| 1. 在经济发展中，各民族同舟共济，相互扶持。 |  |  |  |  |  |
| 1. 我认为熟悉的跨民族群体不会故意触犯我的民族禁忌 |  |  |  |  |  |
| 1. 相对于陌生群体，我更能理解熟悉的其他民族的独特文化。 |  |  |  |  |  |
| 1. 当其他民族使用独特的民族语言时，我相信这仅仅是因为他们民族语言更熟练。 |  |  |  |  |  |
| 1. 我喜欢把本民族文化相关的书籍、视频等分享给熟悉的跨民族群体。 |  |  |  |  |  |
| 1. 我很乐意听其他民族分享他们民族独有的事情（例如文化风俗等）。 |  |  |  |  |  |
| 1. 我愿意与其他民族群体深入地交流各自民族的文化习俗。 |  |  |  |  |  |
| 1. 我会将本民族和其他民族的不同习俗视为彼此文化特色。 |  |  |  |  |  |
| 1. 我愿意主动学习其他民族独特的文化。 |  |  |  |  |  |
| 1. 相比陌生群体，我更愿意接受熟悉的跨民族文化礼仪。 |  |  |  |  |  |
| 1. 我愿意信任熟悉的他民族群体，因为我们的文化融合更多。 |  |  |  |  |  |
| 1. 如果对其他民族信任程度高的话，人们一般不会排斥跨民族婚姻。 |  |  |  |  |  |
